# Efficacy of the population-based pilot colorectal cancer screening, Csongrád county, Hungary, 2015

**DOI:** 10.3906/sag-1908-79

**Published:** 2020-06-23

**Authors:** Mariann RUTKA, Renáta BOR, Tamás MOLNÁR, Klaudia FARKAS, Daniella PIGNICZKI, Anna FÁBIÁN, Márk GYŐRFFY, Anita BÁLINT, Ágnes MILASSIN, Mónika SZŰCS, László TISZLAVICZ, Ferenc NAGY, Zoltán SZEPES

**Affiliations:** 1 1st Department of Internal Medicine, Faculty of Medicine, University of Szeged, Szeged Hungary; 2 Department of Medical Physics and Informatics, Faculty of Science and Informatics,Faculty of Medicine University of Szeged, Szeged Hungary; 3 Department of Pathology, Faculty of General Medicine, University of Szeged, Szeged Hungary

**Keywords:** Colorectal cancer, colon cancer, screening, immune fecal blood test, TNM stage

## Abstract

**Background/aim:**

In Hungary, a nationwide colorectal screening program is about to be introduced in order to improve the high mortality rate of colorectal cancer (CRC). The aim was to summarize experiences from and assess short-term efficacy of the population-based pilot colorectal screening program in 2015 in Csongrád county, Hungary.

**Materials and methods:**

Asymptomatic individuals between the ages of 50 and 70 with average risk of colorectal cancer participated in the program that was based on the two-step screening method: immune fecal blood test and colonoscopy. The short-term efficacy was assessed as the change in total CRC incidence and initial tumor stage in the screening year (2015).

**Results:**

22,130 individuals were invited to participate, and the participation rate was 46.4%. Immune fecal blood test proved to be nonnegative in 1,343 cases (13%), screening colonoscopy was performed in 766 of them (7.5%). Total colonoscopy was performed in 711 individuals. Based on the reports, adenoma was detected in 358 (50.3%) and malignancy in 42 (5.9%) individuals. In the background population, the incidence of colon cancer was higher (183 vs. 228; P = 0.026) and was diagnosed at earlier stage (P = 0.002), while lymph node involvement was lower in 2015 (48.3% vs. 37.1%; P = 0.049).

**Conclusion:**

The Csongrád county population-based colorectal cancer screening was evidently successful on the short-term considering participation rate, and the changes in CRC incidence and stage, thus its national extension is necessary.

## 1. Introduction

Colorectal cancer (CRC) is the third most common type of cancer in males (746,000 new cases/year, 10.0% of all tumours), and the second most common cancer in females (614,000 new cases/year, 9.2% of all tumours), and it is considered to be the leading cause of death in both genders in the word [1,2].

High incidence of CRC is especially characteristic to those Central European countries where national screening program has not been implemented yet [2], like Hungary with the incidence rate of 84.8 new cases/100,000 residents. Consequently, mortality data should also be emphasized in absence of adequate screening. The situation in Hungary is very unfavourable compared to other European countries with the mortality rate of 42.3/100,000 residents [3–4]. As the precancerous stage can be identified well, and carcinogenesis is slow, sporadic CRC is a tumour type that is suitable for screening and enables performing the proper intervention in time. In respect of screening strategies, it is important to suit the requirements of the healthcare system and financial factors as well [5]. In 2003, the European Council supported the introduction of a so called two-step screening program based on first line use of the detection of occult blood in the faeces (immunochemical faecal occult blood test, iFOBT) [6]. In Hungary, an initiative was started in 2002 to introduce a screening program for colorectal cancer within a national public health program called “Egészséges Nemzetért Népegészségügyi Program 2001-2010” (For a Healthy Nation Public Health Program 2001‑2010) [7]. Before introducing a national colorectal screening program, the Hungarian National Health Insurance Fund (Állami Népegészségügyi és Tisztiorvosi Szolgálat, ÁNTSZ) decided to perform a pilot screening supported by the Social Renewal Operational Program (Társadalmi Megújulás Operatív Program, grant agreement no. TÁMOP 6.1.3.A-13-2013-001) of Hungary in Csongrád county to gather experience and to model expenses and results.

The aim of our study was to summarize our experience with the pilot CRC screening program performed with the Hungarian population in Csongrád county and to evaluate short term efficacy of the program based on its effects on the incidence of CRC. 

## 2. Patients and methods

Population-based preliminary CRC screening in Csongrád county was performed between July 2013 and July 2015. Male and female residents between the age of 50 and 70 who had average risk to colorectal cancer and who had no symptoms or complaints participated in the screening. Individuals were selected centrally and in an organized form, feedback of general practitioners (GPs) regarding the list was considered as well. The residents in the final list received an invitation letter with a written information sheet about the screening which contained basic information about malignant tumours of the large intestine and the rectum in an understandable language. Screening was performed in two steps: iFOBT tests were performed from consecutive defecated stools, then, in case of at least one nonnegative result, colonoscopy was performed as a second step.

Faecal occult blood test, the “first step”

Csongrád county has 60 municipalities and a population of 419,366 individuals. The county has 7 governmental districts (Szeged, Hódmezővásárhely, Makó, Mórahalom, Kistelek, Csongrád, Szentes) providing in area care. Participation of the GPs was voluntary due to the preliminary nature of the investigation. 117 GPs (40.48%) have joined to the screening program with their sections (58, 13, 12, 11, 9, 7, 7 GPs, respectively). Stool samples were examined by the central laboratory appointed by the office of the Chief Medical Officer (Országos Tisztifőorvosi Hivatal, OTH) using iFOBT test cassettes operating based on immunochemistry methods. Test cassettes use an antigen-antibody reaction against the protein component of the human haemoglobin, globin part. These tests are specific to human blood. “Nonnegative” result was considered to be either a positive result or some other colourful reaction on the test cassettes (detection threshold: 20 µg/g). Patients having nonnegative results from either one of two consecutive defecated stools were referred to a gastroenterological (endoscopic) check-up examination. The GPs were informed of the test results.

Colonoscopy, the “second step”

In Csongrád county, so called “mapping colonoscopies” are performed in five regional endoscopic centres. In accordance with the qualitative criteria of colonoscopies, the examinations were performed by gastroenterologist specialists experienced in colonoscopy in these centres. Summarizing and processing of the data in this study were performed in the 1st Department of Internal Medicine, University of Szeged.

Evaluating the effects of screening

Short-term efficacy of the screening was assessed by collecting the newly diagnosed CRC cases during one year in the population and evaluating the clinical stage (TNM) of the cancers. Analysis was performed in the year of screening, from 1st January 2015 to 31th December 2015, while control period was analysed from 1st January 2013 to 31th December 2013 (period before screening). We assumed that the composition of the population did not change considerably during the two years. In respect of the screening year (2015), invitations were sent out in two periods (January and March), iFOBT tests were received and evaluated between January and September, and colonoscopies could be performed from February to November. Histological examination of biopsy samples and samples removed during surgery were completed, as well as pTNM staging of the tumours was performed in the Department of Pathology, University of Szeged in accordance with applicable guidelines [8]. 

Statistical analysis

Statistical data were reported as the mean; with frequencies (n) and percentages (%), when appropriate. Pearson’s chi-squared test or Fisher’s exact test was used to analyse categorical data, whereas independent samples t-test was used in case of continuous data. Statistical tests were performed using R statistical software (R version 3.1.2). Values of P < 0.05 were considered significant.

## 3. Results

Execution and data of the screening program

The list of the invited individuals was made with the suggestions of the participated 117 GPs. All 117 sections consisted of the total of 419,336 residents. Regarding the answers and answering time, the colorectal screening program was divided into two invitation periods. First, a total of 10,431 male and female individuals were invited to the screening between the age of 50 and 70 in January 2015, then 11,699 male and female individuals were also invited between the age of 50 and 70 in a second round in March 2015 [9]. Therefore, a total of 22,130 invitations for colorectal screening were delivered. Those, who accepted the invitation, could go to their GPs’ offices to receive a patients’ package containing two stool containers. The containers were supposed to be sent into the evaluating laboratory with post. In case of at least one nonnegative result, the affected individuals were supposed to attend a colonoscopy in one of the 5 in area care endoscopic centre, where the examination was performed.

The results of the “First step”

**Figure 1:**
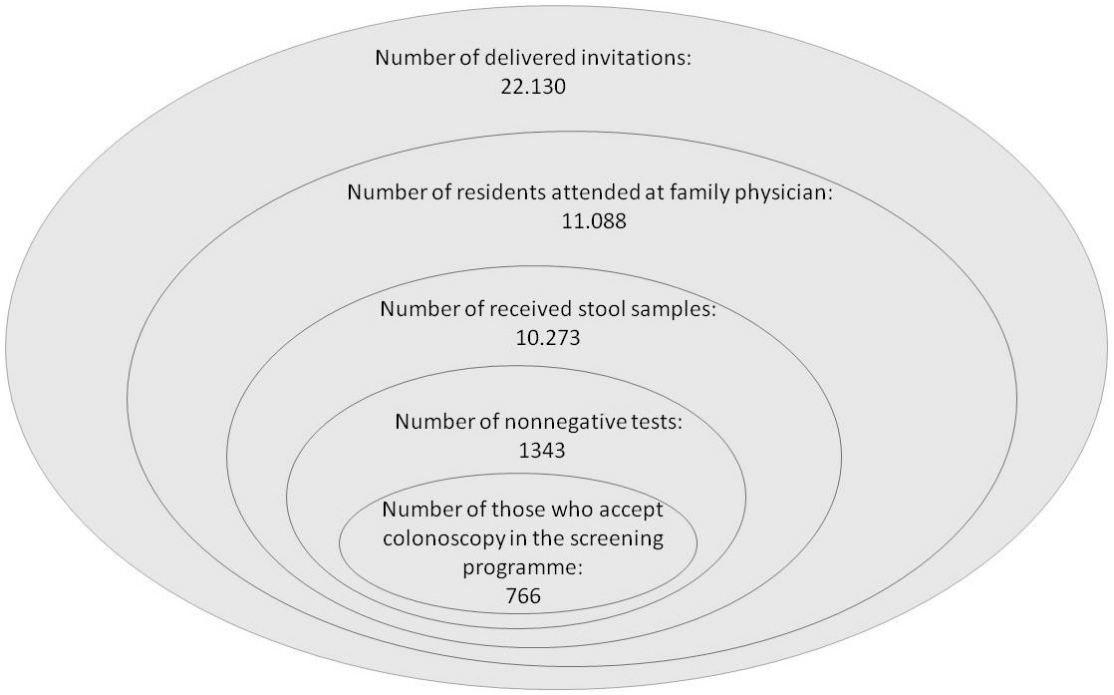
The results of the first step of the Csongrád county screening program.

The total of 22,130 screening invitation were sent out. Altogether, 11,088 residents accepted the invitation and checked-in at the GPs’ offices. All of them received the patient-package – mentioned in the invitation letter – with the two stool containers. 10,273 pairs of evaluable samples were sent to the examining laboratory till 1st September 2015, which means a 46.4% final participation rate in the first step. A total of 1343 (13%) samples were considered to be nonnegative. 766 (7.5%) patients had colonoscopy during the colorectal screening program. The results of the invitation period and the first step of the Csongrád county screening program are described in Figure 1.

*Results of the “Second step”*

Based on the referrals, 786 individuals attended the endoscopic examination and 20 refused to perform endoscopy. Consequently, the total of 766 (97.4%) patients’ colonoscopy was performed between February and November 2015. The colonoscopy results of the 5 endoscopic centres were incorporated in Table. Fifty-five colonoscopies were incomplete because of ineligible bowel preparation, technical difficulties due to previous surgical intervention, the patient’s intolerance or the lack of in patient background (considering polypectomy). Forty-five was performed again to gain a proper description, which finally resulted in 711 complete examinations with the summarized cecal intubation rate of 92.9%. In the following evaluation, we consider these 711 complete colonoscopies as 100% and upcoming percentage of the findings will refer to this total number.

**Table:** Results of processing tables prepared to report examinations performed at the five study sites.

Centre	SZTE	MÁV	HMVH	Makó	Kistelek	Total
Participation of CRC screening	236	267	85	133	65	786
Acceptance of colonoscopy (patients)	236	254	85	132	59	766
Rejection of colonoscopy (patients)	0	13	0	1	6	20
Complete colonoscopies (patients)	219	231	84	125	52	711
Incomplete colonoscopies (patients)	17	23	1	7	7	55
Histology (patients)						
Histology of biopsies:	11	16	0	0	2	29
Low grade dysplasia	8	10	0	0	1	19
High grade dysplasia	3	6	0	0	1	10
Histology of polypectomy specimens:						
Low grade dysplasia	73	68	27	45	15	228
High grade dysplasia	24	34	3	4	13	78
Findings (patients)						
Adenomas:	108	115	30	49	24	335
Low grade dysplasia	81	75	27	45	13	247
High grade dysplasia	27	40	3	4	11	88
No histology needed	9	6	0	5	3	23
Malignant lesions	12	15	0	8	7	42
Other findings (diverticulum, haemorrhoid, hyperplastic inflamed polyp)	90	95	54	63	18	320
Negative findings during endoscopy:	34	14	17	39	4	108
Specialists:	7	2	5	2	4	20

SZTE: Szegedi Tudományegyetem Klinikai Központ I. sz. Belgyógyászati Klinika, MÁV: Vasútegészségügyi Nonprofit Kft., Szeged,

Findings were determined by the number of patients experienced the current most serious finding – based on histological results. Three hundred and fifty-eight patients had adenoma, therefore positive predictive value (PPV) of iFOBT was 50.4%. In respect of grading, 69% of the patients had low grade adenoma as the highest grade adenoma, and 24.6% had high grade dysplasia. Malignant colon tumour was found in 42 cases (5.9%). Summarized PPV of iFOBT regarding adenomas and carcinomas was 56.3%. Colonoscopy showed the absence of abnormal lesions in case of 108 patients (15.2%), therefore in these cases, iFOBT was false positive. In 212 cases (29.8%), other nonmalignant polypoid lesions (hyperplastic or inflammatory polypus), haemorrhoids, inflammatory bowel disease or diverticulosis was confirmed that they could explain the nonnegative faecal blood test.

Long-term efficacy of screening and comparative results

**Figure 2:**
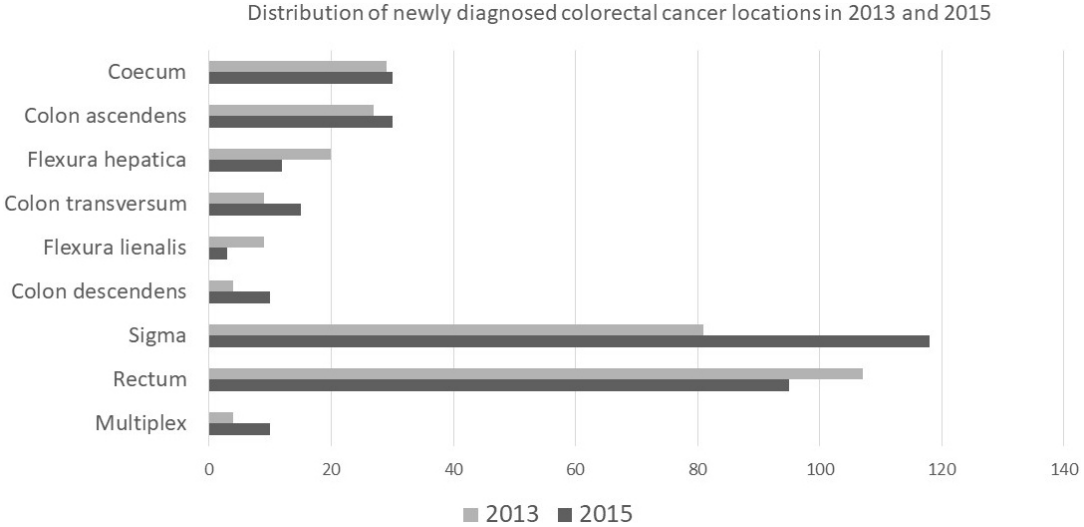
Newly diagnosed tumours during the 2 examined years are summarized in accordance with localization.

The colorectal carcinogenesis is slow and the precancerous stage can be identified well. This brings us the opportunity to work out a suitable screening program, which can provide proper intervention in time, and may save many lives. Newly diagnosed tumours during the 2 examined years are summarized in accordance with localization in Figure 2. The incidence of CRC increased by 11.3% between 2013 and 2015: in 2013, 290 (average age: 67.5, 124 females and 166 males), while in 2015, 323 (average age: 67.5, 140 females and 183 males) new cases were diagnosed. Evaluation of the colon cancer cases showed 183 newly diagnosed malignant tumours in 2013 and 228 new malignant colon tumours in 2015. Comparing the 2 years, a total of 24.5% increase was seen in the recognition of malignant tumours thanks to the screening program. In the year of the screening, significantly more colon cancers were diagnosed (P = 0.013) and it should be emphasized that these tumours were in an earlier stage (P = 0.002). Although the number of recognized carcinomas of almost every segment of the colon was increased at the year of the screening, this tendency was not seen in case of rectum tumours (n = 107 vs. n = 95).

**Figure 3:**
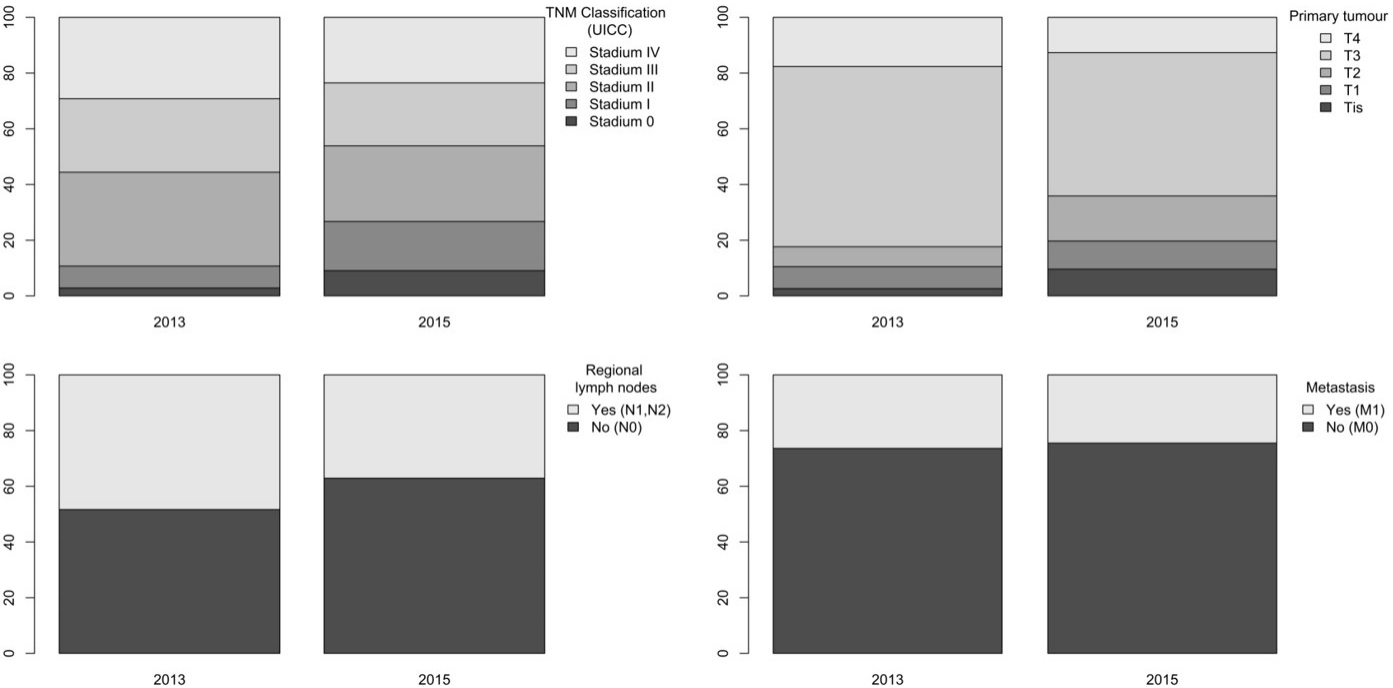
The TNM stages of the colon cancer cases.

**Figure 4:**
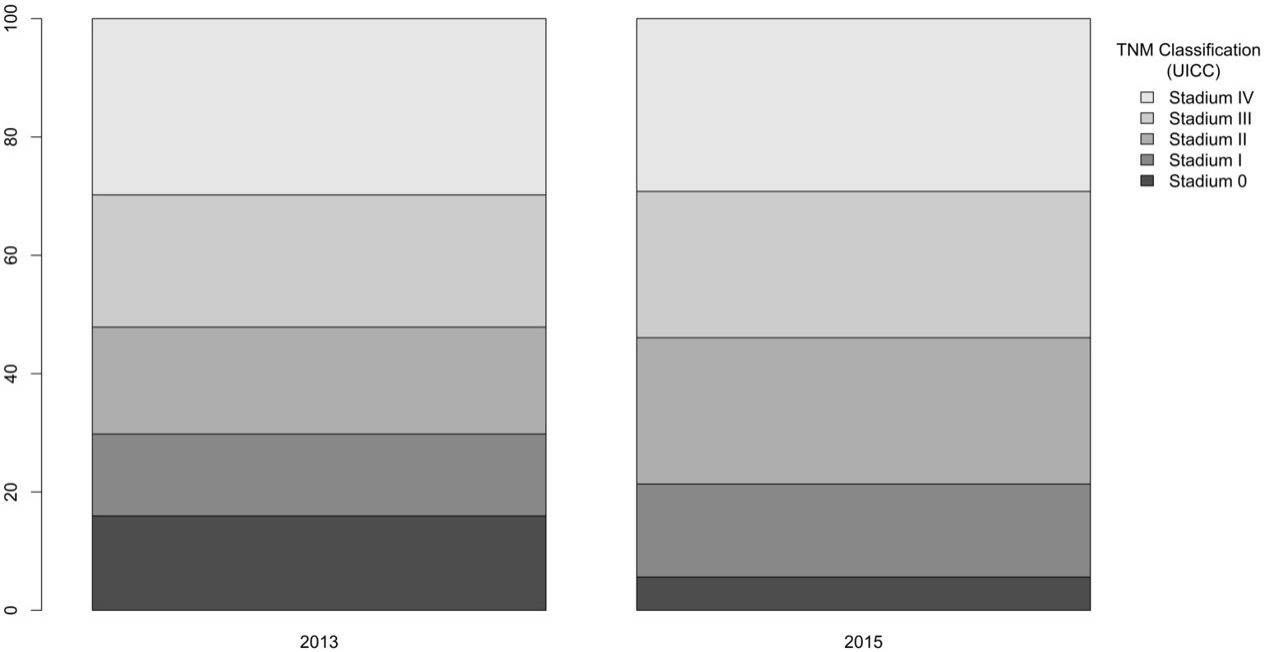
TNM stages of rectum carcinoma cases.

The TNM stage was recorded in case of 272 patients in 2013 and in case of 310 patients in 2015. Based on colon localized TNM staging (based on data of 178 patients in 2013 and 221 patients in 2015), the ratio of “in situ” colon cancer increased from 2.8% (n = 5) to 9.05% (n = 20) from 2013 to 2015, the ratio of stage 1 tumours increased from 7.8% (n = 14) to 17.65% (n = 39). On the contrary, the ratio of stage 2 tumours decreased from 33.7% (n = 60) to 27.15% (n = 60), the ratio of stage 3 tumours decreased from 26.4% (n = 47) to 22.6% (n = 50), and the ratio of stage 4 tumours decreased from 29.2% (n = 52) to 23.5% (n = 52). Evaluation of the T stage (extent of the tumour) showed significant difference between the two periods (P = 0.002), tumours were significantly less extensive in the year of the screening, and significantly less lymph node metastases were found at the time of the diagnosis in the year of the screening (48.3% vs. 37.1%; P = 0.049). No difference was found regarding distant metastases between the two years. Figure 3 summarizes the TNM stage of the colon cancer cases, while Figure 4 shows the TNM stages of rectum carcinoma cases.

## 4. Discussion

Our study evaluates the results and calculates the efficacy of the population-based preliminary colorectal cancer screening program performed in Csongrád county in 2015. The cost-effectiveness is an important point of view. Based on preliminary data, every form of the colorectal cancer screening is cost effective and may save lives. Objective benefits could be determined with 5-10-20 years of follow-up of the screened population comparing mortality data of the screened and not screened population. Such long-term data are available in the United States of America, where the summary of data from 7 cost-effectiveness studies confirmed that cost-effectiveness ratios were 10 000–25 000 dollars per life-year saved [10]. This change would be beneficial for patients and the health care system in a long-term way. 

Survival is the other important aspect beside costs, which has improved by the appearance of adequate screening technologies therapeutic interventions [11]. Between 2006 and 2012 compared to the mid‑1970s, the summarized five-year survival has increased: from 51% to 66% in case of colon cancer and from 48% to 68% in case of rectum cancer [12]. Best prognostic parameter of the outcome of CRC is the TNM stage at the time of the diagnosis. Acting in time is the critical point as early stage CRC patients have a good chance of healing, while the five-year survival of patients with CRC having distant metastasis is under 20% [13]. The 5-year survival of colon tumours having lymph node metastasis increased from 55% to 74%, and in case of rectum tumours, the 5-year survival increased from 45% to 70% [13] resulting in a more promising outcome from patients diagnosed with colorectal cancer with screening. 

It should be emphasized that a decrease in the incidence of the tumour in a certain population occurs only with regular screening [13]. Performing a polypectomy significantly decreases the risk of the development of CRC [14]. The meta-analysis of Shroff et al. [15] summarized data of four randomized controlled trials and confirmed that even a flexible sigmoidoscopy-based screening decreases the mortality and incidence of CRC. Due to this study’s observations, the mortality of sigmoid cancer decreased with 28%, and the mortality in case of distal tumours decreased with 41%. Brenner et al. [16] summarized the results of publications examining CRC incidence rate and different type of screenings. Regarding the findings of these randomised controlled trials and observational studies, flexible sigmoidoscopy and colonoscopy both decreased, but comparison of these 2 types was suggested to be further investigated in respect of proximal tumours. No such randomized studies are available yet, but taking the proximal colon tumours into consideration, we are sure that it is better to do a complete colonoscopy.

Beyond that, preliminary screenings can highlight the potentially improvable points of the future extended screening. Here, we discuss the improvable issues recognized by our preliminary screening. Firstly, financial background must be ensured to guarantee the participation of the GPs, of the endoscopic specialists and the administrational background of the endoscopic centres. In another hand, the target population has to be educated to have a proper insight to the importance of the screening and therefore, to reach more satisfying results. Participation rate in the Csongrád county colorectal screening program was 46.4%. The lack of advertisement and incomprehensive patient education may be laid in its background, so this is a field to get better in. In case of the planned national screening program, composition of the screened population has to be determined considering the risk factors as guidelines recommend and as we have done so. After that, faecal blood test is advisable to be used as step one to avoid unnecessary colonoscopies, which would increase redundantly the burden and costs of the screening. Requirements of the iFOBT-based screening can be found in the consensus guideline of the American Society for Gastrointestinal Endoscopy (ASGE), the American Gastroenterological Association (AGA) and the American College of Gastroenterology (ACG) [17]. Based on this, screening is effective if iFOBT is performed in at least 60% of the invited population, and maximum 5% is lost during the evaluation in the screening laboratory. If these requirements are met, 80% of the patients with positive test results have to be willing to participate in a colonoscopy. Our results from this preliminary study showed a participation rate of 46,4% which is lower than it would be effective, and we have no data from the data loss in the laboratory. In order to the colonoscopy attendance, 57% of our patients with a nonnegative iFOBT test attended colonoscopy (regarding the numbers of 1343 nonnegative tests and 766 colonoscopies), which is also lower than required in case of effectiveness.

In order to improve compliance of the nation-wide screening, general anaesthesia should be available that can alleviate the inconvenience during colonoscopy. Qualitative parameters of the screening also have to be assured. One of the most basic qualitative endoscopic indicators is the cecal intubation rate, which was 92.9% in the Csongrád county pilot CRC screening, and it met the requirement of the ESGE (European Society of Gastrointestinal Endoscopy) [18]. No reliable information was available regarding other important indicators such as the duration of the examination, the rank of practiced examiners or uniformly administered bowel preparation scale. The exact number of the findings and performed interventions were also missed regarding the fact that the patients were labelled only by the most serious findings. Therefore, a standardized registry should be established and filled out by skilled administrators. With respect of the findings, an Italian screening program showed that 30.2% of the removed tumours were high risk adenomas and the ratio of CRC was 3%, in Slovenia, these ratios were 25.16% and 2.16%, while in France, the ratios were 19.6% and 7.5%, respectively [19]. In case of the screening program in Csongrád county, adenoma detection rate was 50.4% with a CRC detection rate of 5.9%. These high percentages are considered to be the consequence of the lack of previous screenings. 

In addition, primer preventive methods should also be emphasized starting with health education in the population at an early age and with informing the population about the risk factors of developing colorectal cancer. Furthermore, polypectomy and postpolypectomy follow-up brings the opportunity to further decrease mortality. The National Polyp Study performed in the United Kingdom studied 2602 patients having one or more polypectomies. The median follow-up of 16 years, while the mortality of CRC has decreased with 53% compared with the general population [20]. Therefore, the triumvirate of prevention, regular screening and follow-up are the basic points of quality care, which can result in the fact that the incidence and mortality of CRC is gradually decreasing for years in those countries which have already introduced a nation-wide screening.

### 4.1. Limitations of the study

Regarding the preliminary nature of the investigation, the participation of the GPs was voluntary. The endoscopic and administrative burden of the screening program was in addition to the daily routine tasks and without financial compensation. This resulted in the fact that Szentes was not able to contribute to the endoscopic data providing from its 7 GPs section, and therefore, 5.5% of the data was lost. Free choice of physicians also contributed to data loss, as patients had the opportunity to attend the colonoscopy in other clinical offices or in private practices besides the appointed physicians. Thus, participation rate in the second step cannot be exactly quantified although it would be an important qualitative parameter that could be potentially improved. Some examination offices only registered the most serious adenoma grades without the exact number of the lesions or the less serious findings.

In conclusion, leading causes of death should be well-screened in any cases, where there is a possibility to recognize the tumour in precancerous or early stage in order to treat them effectively with available interventions, and to encourage the target population for participate. Preliminary screening is useful and instructive in respect of the management of the screening for every country planning any kind of screening. Furthermore, its data can highlight the potentially insufficient or improvable points before putting a nation-wide screening into practice. However, financial issues and administrational background have to be appropriate for being successful.

We encourage every country to introduce any kind of available screening in case of frequent malignant tumours. Hungary is a good example as we worked out a successful preliminary screening, based on which we have already started our nation-wide screening. The two-step CRC screening is specifically recommended as this is beneficial in every considerable aspect.

## Acknowledgments/disclaimers

The study was registered with Regional Human Biomedical Research Ethics Committee, with the identifier 4173. The screening was supported by the Hungarian National Health Insurance Fund (ÁNTSZ) and the Social Renewal Operational Program (TÁMOP) of Hungary (grant agreement no. TÁMOP 6.1.3.A-13-2013-001).
